# Parental age and birth defects: a sibling study

**DOI:** 10.1007/s10654-021-00734-8

**Published:** 2021-03-24

**Authors:** Hans K. Hvide, Julian Johnsen, Kjell G. Salvanes

**Affiliations:** 1 University of Bergen, Bergen, Norway; 2 CEPR, London, UK; 3 University of Aberdeen, Aberdeen, UK; 4 SNF-Centre for Applied Research at NHH, Bergen, Norway; 5 FAIR, Bergen, Norway; 6 Norwegian School of Economics, Bergen, Norway; 7 IZA, Bergen, Norway; 8 HCEO, Bergen, Norway

**Keywords:** Birth disorder, Congenital anomalies, Parental aging, Stillbirth

## Abstract

Higher parental age at childbearing has generated much attention as a potential risk factor for birth disorders; however, previous research findings are mixed. Existing studies have exploited variation in parental age across families, which is problematic because families differ not only in parental age but also in genetic and environmental factors. To isolate the effects of parental age, holding many genetic and environmental factors constant, we exploit the variation in parental age within families and compare outcomes for full siblings. The study data were retrieved from the Medical Birth Registry of Norway, which covers the entire population of births in Norway over an extended period (totaling 1.2 million births). Using variation in parental age when siblings were born, we find large and convex effects of increased parental age on the increased risk of birth disorders. To facilitate comparison with the existing literature, we also estimate the effects of parental age using variation in parental age across families and find that the effects are substantially weaker. We conclude that the existing literature may have underestimated the negative effects of parental aging on adverse offspring outcomes.

**Supplementary Information:**

The online version contains supplementary material available at 10.1007/s10654-021-00734-8.

## Introduction

Parental age at childbearing has increased across the Western world over the last several decades and generated much attention as a potential risk factor for adverse offspring outcomes. These include stillbirth [1], low birth weight [29], birth defects [18, 21, 34], preterm birth [11, 36], suicide risk [5], schizophrenia, and autism [28].

This paper examines the effect of parental aging on birth defects. There are several biological mechanisms why higher parental age could lead to higher risks for congenital anomalies—for example, paternal mutations, increased incidence of aneuploidy, and accumulation of environmental exposures over time [9, 31, 35]. There are also possible modifiers, including a healthier lifestyle with age or access to better prenatal health care.

The findings from the extant literature are mixed and inconclusive on whether higher parental age is associated with a higher risk of birth defects [2, 12, 17, 18, 24, 34, 37]. For the most part, these studies apply cohort and case–control designs, where the source of age variation is that the age of parents differ between families. However, a correlation between a higher risk of birth defects and parental age across families does not isolate the effect of parental age per se. This is because birth defects may correlate with difficult-to-measure genetic variables and environmental factors such as socioeconomic background [8].

To isolate the effects of parental age and reduce the impact of genetic and environmental confounders, we compare outcomes for full siblings, where the source of parental age variation is the same parents getting older. Many genetic and environmental factors are thus held constant. The sibling design seems well suited for analyzing the effects of parental age on birth disorders, as time-varying confounders such as parental attention and sibling interaction would not have had an impact at birth.

We employ data from the population-wide Medical Birth Registry of Norway (MBRN). This dataset covers the entire population of births in Norway over more than 30 years—in all, about 1.2 million births. The main analysis focuses on within-family variation in parental age. However, to facilitate comparisons with the existing literature, we also analyze offspring risk using a between-families (cohort) approach.

Existing literature (cited in the third paragraph) have typically attempted to identify the independent effects of mothers’ age and fathers’age on offspring outcomes. This is not feasible with a standard sibling design, as the ages of the mother and father are perfectly collinear. To approach this issue, we split families into subgroups defined by the age difference between the mother and the father and conduct separate analyses for the different subgroups.

Finally, most studies on congenital anomalies have been unable to capture stillbirths and have likely excluded a significant proportion of anomalous fetuses [12]. We therefore include stillbirths in the analysis. The extant literature, using cohort or case–control designs, has generally found a higher risk of stillbirth with advanced parental age [20].

## Materials and methods

### Study sample

The study data were retrieved from MBRN, which contains the full population of births in Norway from 1967 onward. MBRN provides information on the year of birth for both parents, along with detailed information on the health status of the child at birth. MBRN uses the International Classification of Diseases (ICD), a global standard for classifying diseases maintained by the World Health Organization. We limited our study to births in the period 1967–1998 because MBRN consistently used the ICD-8 classification system for birth disorders during this period. In an appendix analysis (see Fig. 6 in online Appendix 1), we also included births later than 1998, where MBRN used the ICD-10 classification system.^1^

We limited our study to singleton births and excluded children whose parents’ average age was less than 20 years or above 49 years (2.48 and 0.01% of all births, respectively). We excluded the first group because our focus is on the effects of parental aging rather than risk factors surrounding teenage births. We excluded the second group because it contains an insufficient number of births to obtain precise estimates for the 50+ average age category.^2^ We further restricted the sample to children who had at least one full sibling born within this specified range of average age. We dropped the 0.8% of children in the register whose fathers were unknown. The final sample comprised 1,230,070 births in 514,282 mother–father pairs. The average and the median number of children for each mother–father pair are 2.4 and 2, respectively.

### Measurements

All maternity wards in Norway measure outcomes for children using ICD-8 after birth and, subsequently, report these outcomes to MBRN. ICD-8 contains 20 main categories of congenital malformations at the three-digit level. Broadly, these categories contain birth disorders of the limbs and skeleton, as well as the nervous, circulatory, respiratory, digestive, visual, and auditory systems (the categories are listed in online Appendix Table 4).

We created a dummy variable that equals one if a child has at least one disorder, this variable is the focus of the main analysis. We also report the results for the seven most common ICD-8 subcategories of birth disorders. Furthermore, we report the results for infant mortality, defined as miscarriages (after week 12), perinatal mortality (late fetal death or death of a newborn up to 1 week postpartum), and neonatal mortality (newborn death occurring within 28 days postpartum). MBRN contains full information on miscarriages after week 12, stillbirths, and postpartum mortality.

In additional analyses, we report the results for low birth weight (defined as birth weight less than 2500 grams), very low birth weight (defined as birth weight less than 1500 grams), preterm birth (defined as gestational age less than 36 weeks), and low Appearance, Pulse, Grimace, Activity, and Respiration (APGAR) score (defined as APGAR score five minutes after birth < 3). The APGAR score is used to evaluate the health of a newborn on a scale of 0–10.

### Statistical analysis

To analyze the effects of our main predictor variable, average parental age, on birth outcomes of children, we compared outcomes for full siblings. We implemented the sibling design using variations of ordinary least squares regression models with family fixed effects. The family fixed effect enters the models as a family-specific intercept. This intercept absorbs the effects of genetic and environmental factors that are constant over time within the family. The inclusion of the family-specific intercept implies that the models identify the effects of parental age solely from within-family differences in outcomes across siblings. We also controlled for the birth year of each child with yearly fixed effects to accommodate population-wide trends. As it is reasonable to assume no crossover effect between siblings— that is, the parent’s age when one sibling is born does not have a causal effect on the birth outcomes of other siblings, we obtained estimators that can be interpreted as causal effects of parental aging [27]. In online “Appendix 2”, we depict the assumed causal structure by a directed acyclic graph and illustrate how the sibling design deals with unobserved genetic and environmental factors.

To estimate the effect of parental aging, we first followed the approach in the existing literature and categorized (average) parental age into six bins: 20–24, 25–29, 30–34, 35–39, 40–44, and 45–49. Then, we estimated the within-family effects of parental aging by ordinary least squares with the six bin dummies as the predictor variables. Our reference category was 30–34, implying that the coefficient on each of the other bins can be interpreted as the change in the likelihood of observing the given outcome if parental age is within the given age bin compared to the 30–34 bin.^3^ Multiple testing as a result of categorization increases the likelihood of false discoveries; thus, we adjusted the p-values and confidence intervals for multiple hypothesis testing using the false discovery rate method [3]. Furthermore, we report the results from F-tests, which jointly tests for an effect of parental age.^4^

While categorizing average parental age into bins provides easily interpretable coefficient estimates, a drawback is that this approach assumes homogeneity of risk within age categories [4].^5^ This means that families in which all the children are born within the same 5-year band will not contribute to the identification of the coefficients.

Our second approach was to apply a regression spline model [16, 22, 23]. This approach allowed us to estimate the effect of parental aging within the age categories. We used linear parental age splines with knots at 25, 30, 35, 40, and 45 and obtained one coefficient estimate for each age interval, which should be interpreted as the linear effect of increased parental age within that age interval. As in the categorical analysis, we included family and birth year fixed effects.

The extant literature on the effects of parental age typically attempts to identify the independent effects of mothers’ and fathers’ age. To identify the separate effects of mother or father aging is not possible in a standard sibling design, as the aging of the mother and the aging of the father are perfectly collinear. To deal with this issue, we split families into subgroups according to the age difference between the mother and the father, and conducted separate analyses for each subgroup. Similar to a method suggested by Stene and Stene [30], if the effects of fathers’ aging are increasing and convex (i.e., increasing at an increasing rate), then we would expect the aging effects for children with an old father relative to the mother to be stronger than for families where the parental ages are more similar. If the effects of mothers' aging are increasing and convex, we would expect the aging effects for children with an old mother relative to the father to be stronger than for families where the parental ages are more similar.

Our study sample is large (about 1.2 million births), but the sibling approach requires that we estimate a large number of family-specific fixed effects (there are about 500,000 unique mother–father pairs), which means that we have limited power, especially when analyzing rare outcomes such as subcategories of birth disorders. Therefore, in part of the analysis, we bundled younger age groups together such that the reference category became 20–34 and used a regression spline model with knots at 30 and 40. These models come with additional statistical power at the cost of a less flexible functional form. In our tables, we add stars to coefficients that are statistically significant at the 1, 5, and 10% level.

Our analysis focused on within-family variation in parental age. To facilitate comparisons with the existing literature, we also analyzed the effects of parental age using a between-families (cohort) approach for the main outcomes. We performed all data management and analyses using Stata Version 16.1. The standard errors are adjusted for within-parents correlation. “Appendix 3” provides the Stata syntax for the main results.

## Results

**Fig. 1:**
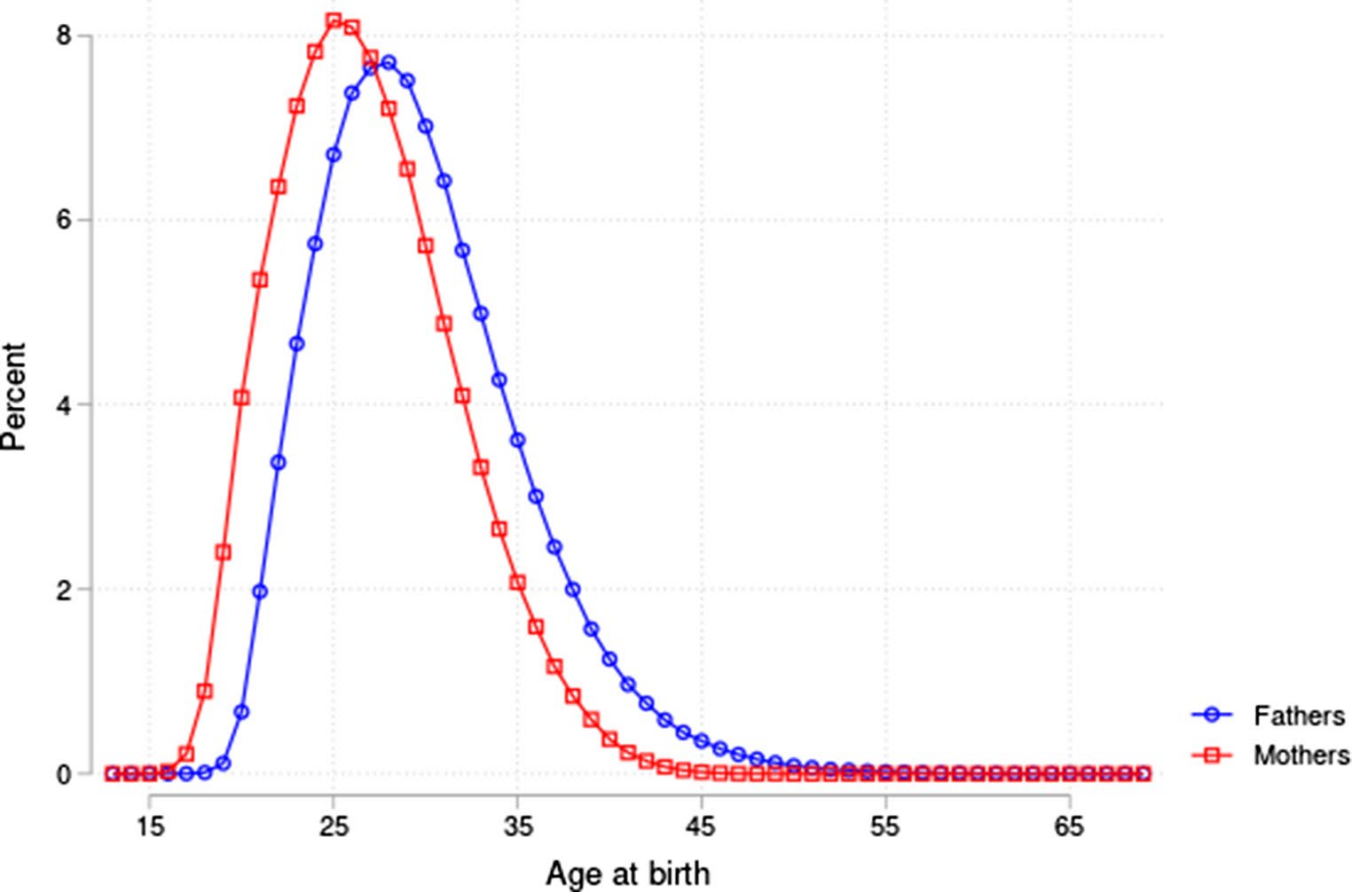
The distribution of age at birth for mothers and fathers in our analysis sample. *Data source*: Norwegian Medical Birth Registry, 1967–1998

32,855 children in our sample were diagnosed with at least one birth disorder, and there were 6331 cases of infant mortality. Of the total 514,282 families (unique mother–father combinations), 35,631 (about 7%) had at least one child with a birth disorder or who experienced infant mortality. Table 1 provides additional descriptive statistics at the parent and offspring levels. The incidence of both congenital malformations and infant mortality was found to be higher among children born to older parents. The incidence of malformations was 3.5% in the age category 45–49 compared to 2.8% in our reference category 30–34. For infant mortality, the incidence was 1.0% in the age category 45–49 compared to 0.4% in our reference category 30–34. For parental characteristics, we first noted that the average age gap between fathers and mothers was increasing with average parental age: 11.5 years in the average age category 45–49 compared to less than 3 years in the reference category 30–34. Looking at completed years of education (measured at age 45), mothers in the older age categories have more years of education, but the same pattern was not found for fathers. Fathers in the older age categories do, however, have significantly higher income measured at age 45. Looking at fathers’ age and health characteristics at age 18 (from military records), we saw no clear pattern between age category and fathers’ characteristics. Figure 1 provides the distribution of fathers’ and mothers’ age at the birth of their offspring. As expected, fathers were slightly older on average than mothers.

**Table 1:** Descriptive statistics of child and parents by average parental age. *Data sources*: Norwegian Medical Birth Registry, 1967–1998, tax registers, education registers, and military records

	Average parent age					
	20–24	25–29	30–34	35–39	40–44	45–49
	(N = 310,361)	(N = 487,744)	(N = 303,872)	(N = 104,922)	(N = 20,841)	(N = 2330)
*Child*						
Boy	0.52	0.51	0.51	0.51	0.52	0.52
Malformation	0.0246	0.0265	0.0282	0.0285	0.0306	0.0348
Infant mortality	0.0066	0.0047	0.0044	0.0051	0.0066	0.0103
*Mother*						
Age at birth	21.57	25.92	30.35	34.53	38.01	40.51
Birth year	1956.35	1955.92	1954.44	1951.13	1946.77	1942.75
Years of education	− 0.000	− 0.003	− 0.001	0.008	0.046	0.214
	(N = 99,325)	(N = 155,576)	(N = 102,528)	(N = 34,499)	(N = 5708)	(N = 490)
*Father*						
Age at birth	23.88	28.49	33.48	38.86	45.01	52.01
Birth year	1954.05	1953.35	1951.31	1946.81	1939.78	1931.24
Years of education	− 0.001	0.003	− 0.012	0.027	− 0.004	0.015
	(N = 104,234)	(N = 159,448)	(N = 99,585)	(N = 30,019)	(N = 4144)	(N = 181)
Income	328.36	− 401.84	− 437.80	2120.80	1710.62	10 418.50
	(N = 107,022)	(N = 162,900)	(N = 101,471)	(N = 30,791)	(N = 4338)	(N = 194)
Height	− 0.001	0.019	− 0.003	− 0.050	− 0.118	− 0.204
	(N = 44,513)	(N = 69,606)	(N = 43,647)	(N = 14,290)	(N = 3070)	(N = 343)
AFQT	0.001	0.001	0.003	− 0.016	− 0.005	− 0.067
	(N = 41,103)	(N = 64,213)	(N = 40,250)	(N = 13,763)	(N = 2825)	(N = 321)
Health	− 0.004	− 0.002	0.004	0.006	0.026	− 0.059
	(N = 33,516)	(N = 52,158)	(N = 32,757)	(N = 11,128)	(N = 2278)	(N = 248)
No health issues	− 0.000	− 0.001	0.001	0.002	0.007	− 0.011
	(N = 33,516)	(N = 52,158)	(N = 32,757)	(N = 11,128)	(N = 2278)	(N = 248)

This table reports descriptive statistics of children and parents. Income and years of education measured at age 45. Income, years of education, height, AFQT, Health and No health issues, are normalized to zero within each (parental) birth cohort, to adjust for differences across birth cohorts

*AFQT* armed forces qualification test score

Table 2 reports the results from the sibling design approach for our main outcomes: birth defects and infant mortality. Columns (1) and (3) report estimates from the model with categorical age variables. The F-test shows that categories of parental age are jointly significant at the 1% level for both birth defects and infant mortality. Further, there is a clear and nonlinear effect of increasing parental age on the outcomes: Children born to parents with an average age of 45–49 were 1.9% points more likely to have a birth defect and 0.8% points more likely to suffer infant mortality compared to children born to parents with an average age of 30–34 (the reference category). These are large effects given that the incidence of birth defects is 2.7% and the incidence of infant mortality is 0.5% in our sample.

**Table 2:** Effect of average parental age on congenital malformations and infant mortality. *Data source*: Norwegian Medical Birth Registry, 1967–1998

	Congenital malformation		Infant mortality	
	OLS	Regression spline	OLS	Regression spline
	(1)	(2)	(3)	(4)
20 ≤ age < 25	0.07	−0.01	0.01	−0.07***
	[−0.17,0.30]	[−0.12,0.10]	[−0.09,0.11]	[−0.12,−0.02]
	(0.591)	(0.882)	(0.879)	(0.009)
25 ≤ age < 30	−0.05	−0.01	−0.07	−0.01
	[−0.24,0.13]	[−0.12,0.09]	[−0.16,−0.03]	[−0.06,0.04]
	(0.591)	(0.805)	(0.161)	(0.742)
30 ≤ age < 35		0.08		0.00
	Excluded	[−0.03,0.18]	Excluded	[−0.05,0.05]
		(0.171)		(0.8736)
35 ≤ age < 40	0.18	0.02	0.05	−0.02
	[−0.09,0.44]	[−0.05,0.07]	[−0.09,0.19]	[−0.08,0.03]
	(0.203)	(0.767)	(0.525)	(0.383)
40 ≤ age < 45	0.46*	0.19*	0.15	0.11**
	[0.09,1.01]	[−0.00,0.38]	[−0.16,0.45]	[0.02,0.20]
	(0.099)	(0.056)	(0.349)	(0.022)
45 ≤ age < 50	1.85***	0.47*	0.79**	−0.13
	[0.66,3.05]	[−0.08,1.03]	[0.11,1.47]	[−0.41,0.14]
	(0.002)	(0.093)	(0.023)	(0.341)
F-test of joint significance	5.37 (0.000)		6.57 (0.000)	
N	1,230,070			

This table reports results from an OLS regression (columns (1) and (3)) and a regression spline (columns (2) and (4)). The model specifications include birth year and family fixed effects. 95% confidence interval (CI) in brackets and *p*-values in parentheses

The CIs and *p*-values in columns (1) and (3) have been adjusted for multiple hypothesis testing using the false discovery rate method. Coefficients and CIs are multiplied by 100. Coefficients in columns (1) and (3) indicate the percentage point change in likelihood of the given outcome for the given age category relative to the reference age category 30–34. The coefficients in columns (2) and (4) indicate the change in likelihood of the given outcome when average parental age increases with 1 year within the given age range. Congenital malformation is an indicator equal to one if the child had at least one congenital malformation of any sort. Infant mortality is an indicator equal to one if the child was stillborn or dead within 28 days of birth

*p < 0.1, **p < 0.05, ***p < 0.01

Columns (2) and (4) report results from the regression spline approach (Table 2). For birth defects, we estimated a positive linear effect of increasing parental age on birth defects for births to older parents. When the average age of the parents was 40–44, increasing the average parental age by 1 year increased the likelihood of birth defects by 0.2% points. When the average age of the parents was 45–49, increasing the average parental age by 1 year increased the likelihood of birth defects by 0.5% points. These coefficients are only statistically significant at the 10% level, but, like the results from the categorical model, indicate that parental age has a nonlinear effect on the likelihood of birth defects. For infant mortality, we found that when the average age of the parents was 40–44, increasing the average parental age by 1 year increased the likelihood of birth defects by 0.1% points. Within the age category 45–49, we estimated a negative but statistically insignificant and very noisy effect of parental age on infant mortality. To increase power in the spline regressions, we estimated the effect of parental age within wider age bins. The results, reported in online Appendix Table 5, showed that within the age bin of 40–49, increasing average parental age by 1 year increased the likelihood of birth defects by 0.2% points. This result is statistically significant at the 5% level.

**Fig. 2:**
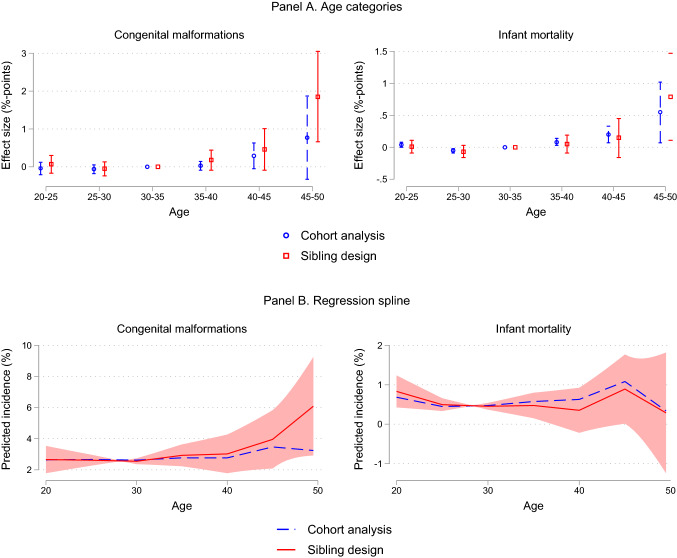
The effect of average parental age on congenital malformations (left) and infant mortality (right). *Notes*: Panel A plots the coefficients and 95% confidence intervals (CI) from an OLS regression of the specified outcome on five indicator variables for categories of average parental age (20–24, 25–29, 35–39, 40–44, and 45–49). The coefficients and CIs are multiplied by 100 and indicate effect size in percentage points. Effects are relative to the reference category of average parental age being 30–34. The red squares indicate coefficients from an OLS regression including family fixed effects, while the blue circles indicate coefficients from an OLS regression without a family fixed effects term. All regressions control for the child’s year of birth. Confidence intervals are adjusted for multiple hypothesis testing using the false discovery rate method. Panel B plots the linear relationship between average parental age and the specified outcome estimated from a regression spline approach allowing for separate linear relationships within each of the age bins 20–24, 25–29, 30–34, 35–39, 40–44, 45–49. The y-axis indicates predicted incidence in percent. The red solid lines indicate our preferred sibling design, while the blue dashed lines indicate a cohort analysis. The red shaded area in Panel B is a 95% confidence interval for the conditional mean prediction from the sibling design, i.e. the red line. *Data source*: Norwegian Medical Birth Registry, 1967–1998

We illustrate the main findings from Table 2 in Fig. 2. Panel A (red squares) shows the effect of parental aging obtained from the model with categorical age variables, and Panel B (red squares) shows the effect of parental aging when using regression splines. Figure 2 also includes estimates based on models without a family fixed effect (blue circles) (i.e., a cohort analysis). Both panels highlight stronger detrimental effects of parental aging using a sibling design than what is obtained by a cohort analysis, especially for congenital anomalies. For example, in Panel B, for congenital anomalies, the continuous effect of increased parental age is flat until the age bins 40–44 and 45–49, after which there is a positive and increasing effect. The sibling design gives a steeper trajectory than the cohort analysis. For infant mortality, the picture is more unclear.^6^ In Panel B we include a 95% confidence interval for the conditional mean prediction (red shaded area). This confidence interval, in the statistical literature also referred to as the confidence interval of the fitted value, reflects the sum of uncertainty about the constant term and the slope parameters of the spline regression [33].

In Table 3, we investigated subcategories of birth defects. Panel A provides results from the categorical model. The F-test shows that categories of parental age are jointly significant at the 1% level for other congenital anomalies of the musculoskeletal system and for congenital syndromes affecting multiple systems. Furthermore, the categories of parental age are jointly significant at the 5% level for congenital anomalies of heart and congenital clubfoot. However, Panel A shows that we lack the statistical power to investigate the effects of each separate age category (after adjusting for multiple hypothesis testing). In Panel B, we increased power by expanding the reference category from 30–34 to 20–34. We found a statistically significant and increasing effect of parental age on clubfoot, other congenital anomalies of the musculoskeletal system, and congenital syndromes affecting multiple systems. Having parents in the 45–49 age category increases the likelihood of being born with these types of birth defects by 0.43, 0.26, and 0.58% points, respectively. These are large effects given that the incidence of these three categories in the sample are 0.58, 0.12, and 0.14%, respectively.^7^

**Table 3:** Effects on separate subcategories of malformations

	Heart	Palate/lip	Genital	Clubfoot	Limbs other	Musculo-skeletal other	Multiple systems	Other
	(1)	(2)	(3)	(4)	(5)	(6)	(7)	(8)
Panel A. Reference category is 30 ≤ age < 35								
20 ≤ age < 25	0.02	− 0.03	0.03	− 0.03	− 0.04	0.04	0.04	0.00
	[− 0.09,0.13]	[− 0082,0.76]	[− 0.48,0.53]	[− 0.15,0.08]	[− 0.16,0.07]	[− 0.03,0.10]	[− 0.04,0.12]	[− 0.09,0.09]
	(0.721)	(0.945)	(0. 921)	(0.559)	(0.473)	(0. 304)	(0.349)	(0.994)
25 ≤ age < 30	− 0.01	− 0.02	0.01	− 0.04	− 0.04	0.02	0.00	− 0.01
	[− 0.05,0.03]	[− 0.56,0.52]	[− 0.11,0.12]	[− 0.15,0.07]	[− 0.16,0.07]	[− 0.01,0.05]	[− 0.03,0.03]	[− 1.99,1.98]
	(0.721)	(0. 945)	(0. 921)	(0.484)	(0. 473)	(0.304)	(0.847)	(0.731)
35 ≤ age < 40	0.05	− 0.00	− 0.00	0.09	0.05	0.03	0.07**	− 0.06
	[− 0.06,0.16]	[− 0.04,0.04]	[− 0.07,0.06]	[− 0.02,0.21]	[− 0.08,0.17]	[− 0.03,0.09]	[0.01,0.13]	[− 0.37,0.25]
	(0.357)	(0.937)	(0.921)	(0.111)	(0. 473)	(0. 304)	(0.032)	(0. 994)
40 ≤ age < 45	0.09	0.04	− 0.08	0.24**	0.07	0.08	0.31***	− 0.07
	[− 0.27,0.45]	[− 0.97,1.04]	[− 1.59,1.42]	[0.01,0.47]	[− 0.12,0.27]	[− 0.02,0.19]	[0.16,0.47]	[− 1.99,1.98]
	(0.637)	(0. 945)	(0. 921)	(0.042)	(0. 473)	(0.105)	(0.000)	(0. 994)
45 ≤ age < 50	0.12	0.26	0.14	0.46	0.21	0.22	0.54*	0.18
	[− 0.51,0.75]	[− 0.63,1.14]	[− 2.47,2.76]	[− 0.11,1.04]	[− 0.35,0.78]	[− 0.11,0.56]	[− 0.04,0.12]	[− 6.02,6.39]
	(0.721)	(0.584)	(0. 921)	(0.116)	(0. 473)	(0.188)	(0.056)	(0. 994)
F-test	2.31 (0.041)	0.75 (0.588)	0.65 (0.659)	2.67 (0.021)	0.59 (0.709)	3.45 (0.004)	8.70 (0.000)	0.95 (0.445)
Panel B. Reference category is 20 ≤ age < 35								
35 ≤ age < 40	0.06**	− 0.10	0.01	0.09**	0.04	0.04**	0.09***	− 0.05
	[0.00,0.12]	[− 0.06,0.04]	[− 0.05,0.07]	[0.01,0.17]	[− 0.09,0.17]	[0.01,0.08]	[0.04,0.14]	[− 0.16,0.05]
	(0.035)	(0.720)	(0.809)	(0.037)	(0.593)	(0.016)	(0.001)	(0.326)
40 ≤ age < 45	0.11	0.02	− 0.06	0.22**	0.05	0.11***	0.35***	− 0.07
	[− 0.03,0.25]	[− 0.07,0.11]	[− 0.39,0.27]	[0.04,0.40]	[− 0.12,0.21]	[0.04,0.18]	[0.20,0.49]	[− 0.21,0.07]
	(0.114)	(0.720)	(0.733)	(0.019)	(0.593)	(0.12)	(0.000)	(0.326)
45 ≤ age < 50	0.15	0.22	0.18	0.43**	0.17	0.26**	0.58***	0.18
	[− 0.17,0.47]	[− 0.39,0.83]	[− 0.79,1.15]	[0.01,0.85]	[− 0.42,0.76]	[0.05,0.48]	[0.14,1.03]	[− 0.18,0.55]
	(0.367)	(0.484)	(0.733)	(0.046)	(0.593)	(0.016)	(0.010)	(0.326)
F-test	2.54 (0.055)	0.84 (0.470)	0.88 (0.450)	3.72 (0.011)	0.36 (0.784)	5.22 (0.001)	11.41 (0.000)	1.52 (0.208)
*N*	1,230,070							

This table reports results from an OLS regression including family and birth year fixed effects. 95% confidence interval (CI) in brackets and *p*-values in parentheses, both adjusted for multiple hypothesis testing using the false discovery rate method

Coefficients (and CIs) are multiplied by 100 and indicate the percentage point change in likelihood of the given outcome for the given age category relative to the reference age category. Columns represent different ICD8 categories of congenital malformations: (1): 746 = Congenital anomalies of heart, (2): 749 = Cleft palate and cleft lip, (3): 752 = Congenital anomalies of genital organs, (4): 754 = Clubfoot (congenital], (5): 755 = Other congenital anomalies of limbs, (6): 756 = Other congenital anomalies of musculoskeletal system, (7): 759 = Congenital syndromes affecting multiple systems, (8): remaining categories not covered by (1)–(7). *Data source*: Norwegian Medical Birth Registry, 1967–1998

*p < 0.1, **p < 0.05, ***p < 0.01

**Fig. 3:**
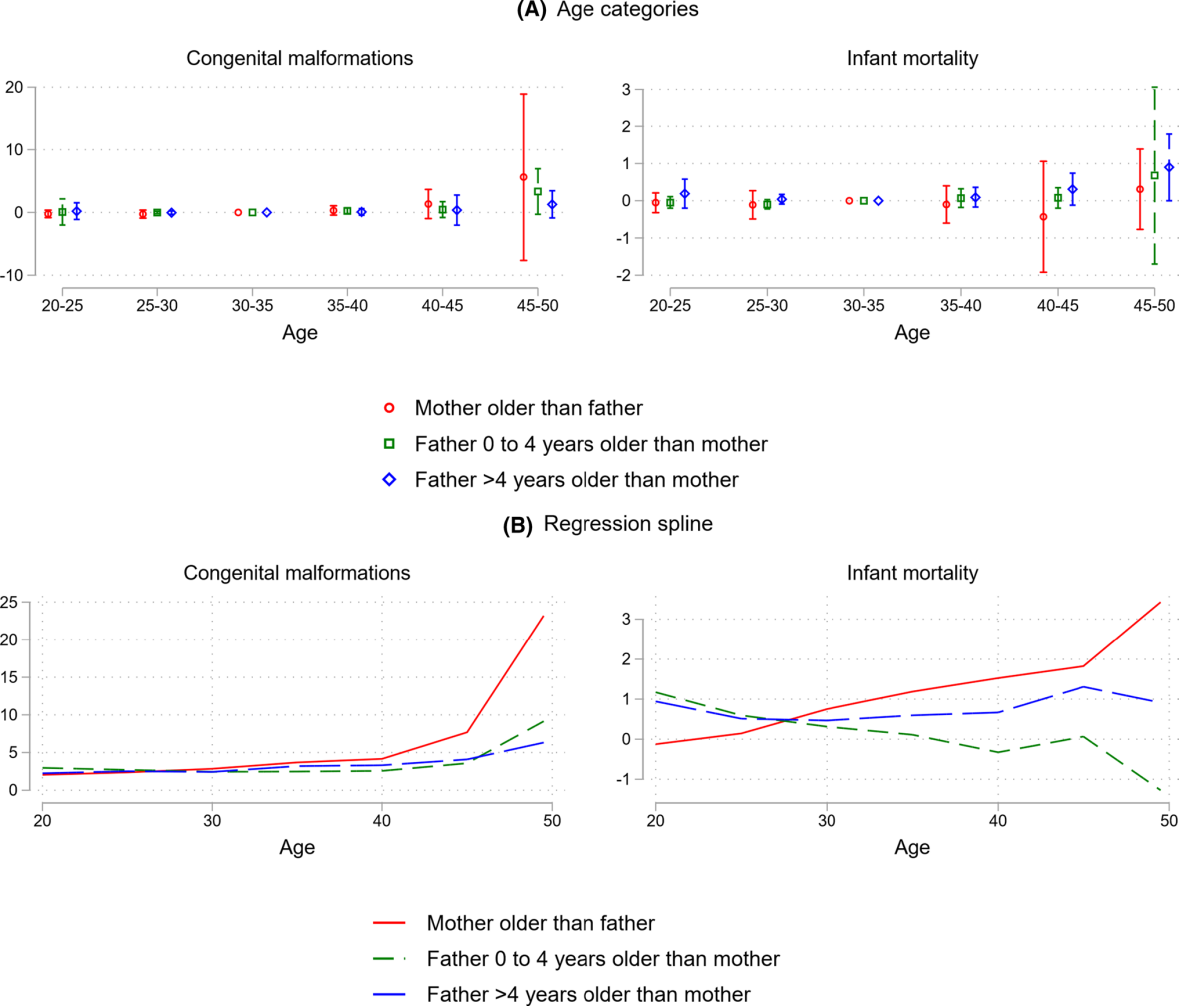
Effects by father–mother age gap. *Notes*: This figure shows the effect of average parental age on congenital malformations (left) and infant mortality (right), contrasting the effect in families in which the mother is older than the father (red) with the effect in families in which father is 0–4 years older than the mother (green) and families in which the father is more than 4 years older than the mother (blue). Panel A plots the coefficients and 95% confidence intervals (CI) from an OLS regression of the specified outcome on five indicator variables for categories of average parental age (20–24, 25–29, 35–39, 40–44, and 45–49). The coefficients and CIs are multiplied by 100 and indicate effect size in percentage points. Effects are relative to the reference category of average parental age being 30–34. All regressions control for family and year of birth fixed effects. Confidence intervals are adjusted for multiple hypothesis testing using the false discovery rate method. Panel B plots the linear relationship between average parental age and the specified outcome estimated from a regression spline approach allowing for separate linear relationships within each of the age bins 20–24, 25–29, 30–34, 35–39, 40–44, 45–49. The y-axis indicates predicted incidence in percent. All regressions control for family and year of birth fixed effects. *Data source*: Norwegian Medical Birth Registry, 1967–1998

In Fig. 3, we split the families into three subsamples according to the age difference between the mothers and the fathers (this difference is constant across births). The red circles and red lines are for the subsample where the mother is older than the father, the green squares and green lines are for where the father is 0–4 years older than the mother, and the blue diamonds and blue lines are for where the father is more than 4 years older than the mother. For congenital anomalies, the effects of parental aging are stronger for the group where the mother is older than the father, suggesting that advanced maternal age could have a stronger influence than paternal age.

**Fig. 4:**
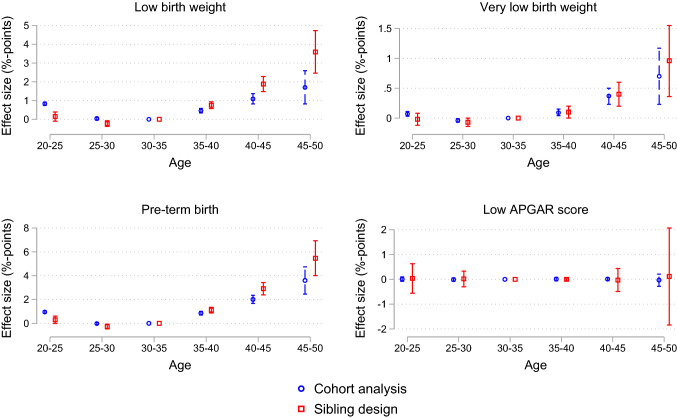
The effect of parental age on low birth weight, very low birth weight, pre-term birth, and low APGAR score: Categorical age variables. *Notes*: This figure shows the estimated coefficients from an OLS regression of average parental age on low birth weight (< 2500 grams), very low birth weight (< 1500 grams), pre-term birth (gestational age < 36 weeks), low APGAR score (APGAR score at 5 min < 3). The figure plots the coefficients and 95% confidence intervals (CI) from an OLS regression of the specified outcome on five indicator variables for categories of average parental age (20–24, 25–29, 35–39, 40–44, and 45–49). The coefficients and CIs are multiplied by 100 and indicate effect size in percentage points. Effects are relative to the reference category of average parental age being 30–34. The red squares indicate coefficients from an OLS regression including family fixed effects, while the blue circles indicate coefficients from an OLS regression without a family fixed effects term. All regressions control for child’s year of birth. Confidence intervals are adjusted for multiple hypothesis testing using the false discovery rate method. *Data source*: Norwegian Medical Birth Registry, 1967–1998

**Fig. 5:**
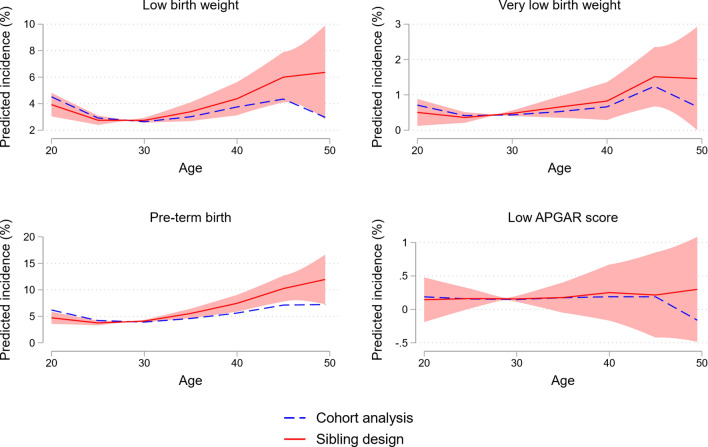
The effect of parental age on low birth weight, very low birth weight, pre-term birth, and low APGAR score: Regression spline. *Notes*: This figure shows estimates from a regression spline approach, plotting the linear relationship of average parental age and low birth weight (< 2500 grams), very low birth weight (< 1500 grams), pre-term birth (gestational age < 36 weeks), low APGAR score (APGAR score at 5 min < 3). The y-axis indicates predicted incidence in percent. The regression spline allows for separate linear relationships within each of the age bins 20–24, 25–29, 30–34, 35–39, 40–44, 45–49. The red solid lines indicate our preferred sibling design, while the blue dashed lines indicate a cohort analysis. The red shaded area is a 95% confidence interval for the conditional mean prediction from the sibling design, i.e. the red line. *Data source*: Norwegian Medical Birth Registry, 1967–1998

In Figs. 4 and 5, we repeated the analyses in Fig. 1 for some additional outcomes, including preterm birth and low birth weight. The same pattern as for congenital anomalies and stillbirth emerged: we found a strong and nonlinearly increasing negative effect of parental age on all outcomes except the likelihood of being assigned a low APGAR score. Furthermore, the sibling analysis suggests stronger detrimental effects of parental aging than the cohort analysis. This is especially the case for the oldest age categories, low birth weight, and preterm birth.

In online Appendix Fig. 6, we augmented the data with all births in Norway from 1999 to 2015. Qualitatively, the results for this larger dataset are similar to the main analysis: the sibling approach revealed a stronger gradient of parental age than the cohort analysis. The confidence intervals were narrower and the difference in the odds ratio between the cohort and the sibling approach was smaller. The latter is likely due to intensified prenatal diagnostics of older parents during the 2000s.

## Discussion

Using data covering all births in Norway over 32 years and employing a sibling design, we found that increased parental age is strongly associated with the increased risk of offspring birth defects and stillbirth. We found similar results for low birth weight and preterm birth. Moreover, the effect of parental aging on adverse birth outcomes appears to be convex: while the 40–44 parental age category had an increased risk relative to the benchmark group, it was vastly exceeded by the risk for the 45–49 parental age category. The first main conclusion of the paper is that there appears to be a strong and convex causal effect of parental aging on the increased risk of children’s adverse outcomes.

Using a cohort analysis, which exploits between-family variation in parental age and is customary in the reviewed literature, we found a weaker association between parental age at birth and increased risk of offspring birth disorders or stillbirth. These findings are consistent with previous studies [20, 25]. The second main conclusion of the paper is that the methods applied by the existing literature may have led to a underestimation of the effects of parental aging on offspring birth defects and stillbirth.

Our study has a few shortcomings, and some of them suggest avenues for further study. First, the sibling approach does not easily extend to analyze the separate effect of mother’s and father’s age, as these two are perfectly collinear within a family. When splitting families into subgroups according to the age difference between the mother and father, the effects of parental aging on birth defects are stronger for the families where the mother is relatively old compared with the father. This suggests that increased maternal age may be more detrimental to offspring outcomes than increased paternal age.

Second, while we can interpret our estimates as causal effects, we only identified the effect of parental age for a specific subset of children: our estimates are based on families with multiple children and might not generalize to singleton families. However, it seems natural to conjecture that the same detrimental effects of aging will be at play for such couples.

Third, the stark contrast in estimated risk patterns between the cohort and the sibling analysis suggests that couples that have children later are positively selected on genetic or environmental factors: while the sibling analysis shows strong effects of parental aging within a given family, the cohort analysis suggests smaller impacts of parental age when comparing outcomes across families. In other words, for a given couple, the decision to postpone childbirth into advanced parental age increases the likelihood of birth defects, but couples that have their first child later are positively selected on average. What are the factors that older parents are positively selected on? A substantial literature suggests that the timing of first birth is correlated with low education level, low income, and religious affiliation Carbone and Cahn [7], Glass and Levchak [14].^8^ However, these correlations are to some extent mechanic: a 21-year old mother could hardly have a college degree, and even with such a degree, she is not likely to have a high labor market income due to lack of experience. To circumvent this problem, we should look at individual characteristics that are not mechanically affected by age at first birth. Table 1 compares younger and older parents based on some of these characteristics—including, the health of the father at age 19 (military records are only available for men), educational attainment for the mother at age 45, and income and education for the father at age 45. These variables are likely to be unaffected by whether a person has children, for instance, at age 25 or age 35. Table 1 reveals some differences; for example, in the 45–49 category, mothers have higher education and fathers have higher income compared to the other groups.^9^ The question of which factors determine the postponing of childbirth remains an important one for further studies.

Fourth, while sibling studies are powerful ways to deal with the selection issues (genetic and environmental confounders) that plague cohort studies, they are not a panacea [8, 10, 15, 19]. One concern is the effect of time-varying factors other than parental age. If these are unaccounted for, the coefficients on parental age will be biased. This bias is especially a problem when studying later-life outcomes such as suicide risk and mental health disorders, where parental time and financial resources during upbringing likely differ for younger and older children [5]. In this study, we examined birth outcomes where such environmental factors are expected to play a lesser role. Another general concern with sibling designs is that misclassification errors may seriously cause bias in the estimates [15]. In the current context, the measurements of infant mortality and birth disorders should be objective and precise in the birth registry, and the misclassification of fathers should be rare so that bias due to misclassification errors should not be a very serious concern.

## Electronic supplementary material

Below is the link to the electronic supplementary material.

(PDF 388 kb)

## Footnotes

1 The ICD is revised periodically and there are major differences between versions ICD-8 and ICD-10 (see WHO [32], https://www.who.int/classifications/help/icdfaq/en/) creating a break in the data that is not easily reconciled. For example, the fraction of children born in 1998 with birth disorders (based on ICD-8) is considerably lower than the fraction of children born in 1999 (based on ICD-10). Moreover, the subcategories of congenital malformations in ICD-8 do not map into subcategories of ICD-10.

2 Our main findings are robust and include these groups (results available upon request).

3 We had to exclude one category from the regression model, as including all the age categories would result in perfect multicollinearity.

4 As this method is commonly used in the literature, we also provide results when estimation is done using conditional logistic regression. Note, however, that there are interpretational issues when using the sibling design and conditional logistic regression in combination [27].

5 Another problem with categorization is that results are difficult to compare between studies that use different age cuts. This critique is less applicable to the present study, as we used the 5-year age cuts that are conventionally used in the literature [13, 17, 34,18,].

6 See online Appendix Table 6 for all results for the cohort analysis.

7 See online appendix Table 7 for results from estimating the model with sibling design and categorical variables using conditional logistic regression.

8 A *New York Times* article from 2018 discusses some of the trends and main findings from this literature.

9 To dig further into this question, we have also compared characteristics of grandparents. However, due to the register data time span, we could not identify a sufficient number of grandparents for births in the highest age categories to perform a meaningful statistical analysis.
