# Supplementary material for: Parental age and birth defects: a sibling study
Source: Eur J Epidemiol. 2021 Mar 24;36(8):849–60. doi: 10.1007/s10654-021-00734-8 (PMC8416834; doi:10.1007/s10654-021-00734-8)
Supplement: Supplementary file 1 — (PDF 388 kb) [file 10654_2021_734_MOESM1_ESM.pdf]

Online appendix for:
Parental Age and Birth Defects: A Sibling Study

Hans K. Hvide
University of Bergen

Julian V. Johnsen
SNF – Centre for Applied Research at SNF

Kjell G. Salvanes
Norwegian School of Economics

Appendix 1. Additional figures and tables

Fig. 6

Including the period 1999 to 2015 in the sample. *Notes:* This figure illustrates on the effects of average parental age on congenital malformations (left) and infant mortality (right) when expanding the sample to include 1999-2015. Panel A plots the coefficients and 95% confidence intervals (CI) from an OLS regression of the specified outcome on five indicator variables for categories of average parental age (20-24, 25-29, 35-39, 40-44, and 45-49). The coefficients and CIs are multiplied by 100 and indicate effect size in percentage points. Effects are relative to the reference category of average parental age 30-34. The red squares indicate coefficients from an OLS regression including family fixed effects, while the blue circles indicate coefficients from an OLS regression without a family fixed effects term. All regressions control for child's year of birth. Confidence intervals are adjusted for multiple hypothesis testing using the false discovery rate method. Panel B plots the linear relationship between average parental age and the specified outcome estimated from a regression spline approach allowing for separate linear relationships within each of the age bins 20-24, 25-29, 30-34, 35-39, 40-44, 45-49. The y-axis indicates predicted incidence in percent. The red solid lines indicate our preferred sibling design, while the blue dashed lines indicate a cohort analysis. The red shaded area is a 95 percent confidence interval for the conditional mean prediction from the sibling design, i.e. the red line. Data source: Norwegian Medical Birth Registry, 1967–2015.

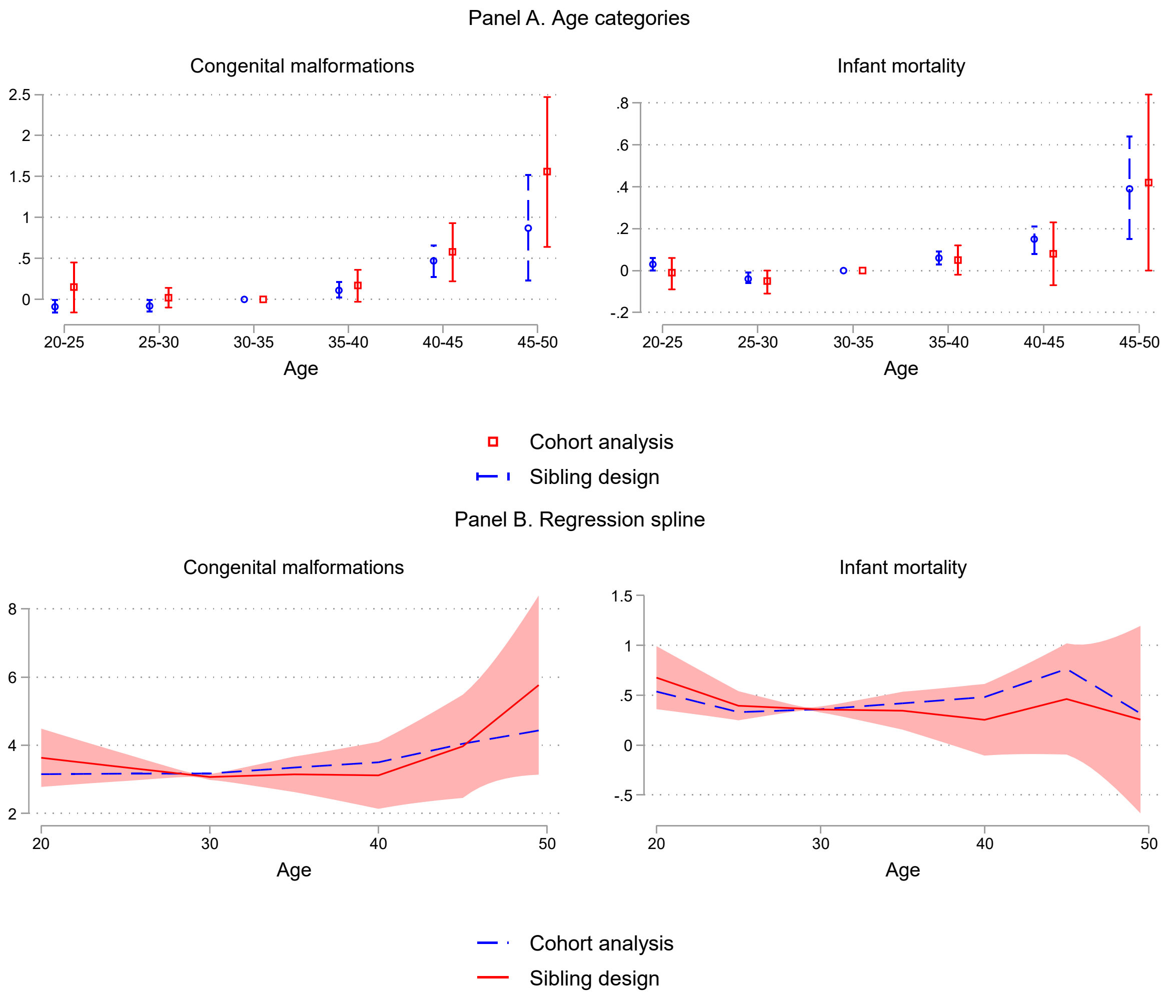

Table 4.
List and incidence of congenital malformations in the 20 main ICD-8 categories.
Data source: Norwegian Medical Birth Registry, 1967-1998.

ICD-8 code	Category	Incidence (%)
740	Anencephalus	*0.007*
741	Spina bifida	*0.038*
742	Congenital hydrocephalus	*0.021*
743	Other congenital anomalies of nervous system	*0.014*
744	Congenital anomalies of eye	*0.017*
745	Congenital anomalies of ear, face and neck	*0.050*
746	Congenital anomalies of heart	*0.212*
747	Other congenital anomalies of circulatory system	*0.032*
748	Congenital anomalies of respiratory system	*0.057*
749	Cleft palate and cleft lip	*0.174*
750	Other congenital anomalies of upper alimentary tract	*0.026*
751	Other congenital anomalies of digestive system	*0.045*
752	Congenital anomalies of genital organs	*0.393*
753	Congenital anomalies of urinary system	*0.057*
754	Clubfoot (congenital)	*0.578*
755	Other congenital anomalies of limbs	*0.647*
756	Other congenital anomalies of musculoskeletal system	*0.116*
757	Congenital anomalies of skin, hair and nails	*0.064*
758	Other and unspecified congenital anomalies	*0.006*
759	Congenital syndromes affecting multiple systems	*0.136*

This table lists the main 20 categories of congenital malformations in the International Classification of Diseases (ICD) version 8, along with the incidence of each category in our study sample.

Table 5
Regression spline with sibling design, dividing average parental age into bins of 20-30, 30-40, and 40-50.
Data source: Norwegian Medical Birth Registry, 1967-1998.

	Congenital malformation	Infant mortality
	(1)	(2)
20≤age<30	-0.01	-0.03
	[-0.11,0.10]	[-0.08,0.02]
	(0.874)	(0.249)
30≤age<40	0.06	0.00
	[-0.05,0.16]	[-0.05,0.05]
	(0.285)	(0.927)
40≤age<50	0.19**	0.06
	[0.03,0.36]	[-0.02,0.14]
	(0.020)	(0.146)
N	1,230,070	

This table reports results from a regression spline. The model specification includes sibling and birth year fixed effects. 95 % confidence interval in brackets and p-values in parentheses.
Coefficients (and CIs) are multiplied by 100 and indicate the change in likelihood of the given outcome when average parental age increases with one year within the given age range. Congenital malformation is an indicator equal to one if the child had at least one congenital malformation of any sort. Infant mortality is an indicator equal to one if the child was stillborn or dead within 28 days of birth.
* $p < 0.1$, ** $p < 0.05$, *** $p < 0.01$.

Table 6

Effect of average parental age on congenital malformations and infant mortality, cohort analysis. Data source: Norwegian Medical Birth Register, 1967–1998.

	Congenital malformation		Infant mortality	
	OLS	Regression spline	OLS	Regression spline
	(1)	(2)	(3)	(4)
20≤age<25	-0.04<br>[-0.13,0.04]<br>(0.602)	0.00<br>[-0.02,0.03]<br>(0.746)	0.04**<br>[0.00,0.08]<br>(0.049)	-0.05***<br>[-0.06,-0.03]<br>(0.000)
25≤age<30	-0.06<br>[-0.14,0.01]<br>(0.281)	-0.01<br>[-0.03,0.01]<br>(0.462)	-0.05**<br>[-0.08,-0.02]<br>(0.28)	0.00<br>[-0.00,0.01]<br>(0.296)
30≤age<55	*excluded*	0.03**<br>[0.00,0.06]<br>(0.036)	*excluded*	0.02***<br>[0.01,0.03]<br>(0.000)
35≤age<40	0.03<br>[-0.09,0.14]<br>(0.646)	-0.00<br>[-0.05,0.05]<br>(0.979)	0.08**<br>[0.03,0.13]<br>(0.42)	0.01<br>[-0.01,0.03]<br>(0.344)
40≤age<45	0.29*<br>[0.05,0.53]<br>(0.092)	0.14**<br>[0.02,0.27]<br>(0.028)	0.20**<br>[0.09,0.31]<br>(0.003)	0.09***<br>[0.02,0.16]<br>(0.007)
45≤age<50	0.77<br>[0.03,1.51]<br>(0.170)	-0.05<br>[-0.50,0.40]<br>(0.826)	0.55**<br>[0.12,0.97]<br>(0.023)	-0.17*<br>[-0.35,0.01]<br>(0.072)
F-test of joint significance	3.17<br>(0.007)		13.56<br>(0.000)	
N	1,230,070			

This table reports results from an OLS regression (columns (1) and (3)) and a regression spline (columns (2) and (4)). The model specifications include birth year fixed effects, but not family fixed effects. 95 % confidence interval (CI) in brackets and p-values in parentheses. The CIs and p-values in columns (1) and (3) have been adjusted for multiple hypothesis testing using the false discovery rate method. Coefficients and CIs are multiplied by 100. Coefficients in columns (1) and (3) indicate the percentage point change in likelihood of the given outcome for the given age category relative to the reference age category 30-34. The coefficients in columns (2) and (4) indicate the change in likelihood of the given outcome when average parental age increases with one year within the given age range. Congenital malformation is an indicator equal to one if the child had at least one congenital malformation of any sort. Infant mortality is an indicator equal to one if the child was stillborn or dead within 28 days of birth.

* $p < 0.1$, ** $p < 0.05$, *** $p < 0.01$.

Table 7
Logit conditional on family fixed effects
Data source: Norwegian Medical Birth Register, 1967–1998

	Malformations									
	Any	ICD8 746	ICD8 749	ICD8 752	ICD8 754	ICD8 755	ICD8 756	ICD8 759	Other	Infant mortality
	(1)	(2)	(3)	(4)	(5)	(6)	(7)	(8)	(9)	(10)
20≤age<25	1.04	1.12	0.86	1.07	0.90	0.94	1.26	1.32	1.01	1.06
	[0.92,1.17]	[0.38,3.32]	[0.14,5.13]	[0.51,2.24]	[0.73,1.11]	[0.76,1.16]	[0.66,2.39]	[0.69,2.52]	[0.81,1.25]	[0.86,1.30]
25≤age<30	0.98	0.98	0.90	1.01	0.89	0.94	1.10	1.04	0.98	0.87
	[0.93,1.04]	[0.81,1.18]	[0.23,3.19]	[0.88,1.16]	[0.76,1.03]	[0.77,1.15]	[0.84,1.45]	[0.82,1.31]	[0.53,1.82]	[0.73,1.03]
35≤age<40	1.06	1.18	0.98	0.97	1.20	1.06	1.36	1.24	0.86	1.15
	[0.95,1.19]	[0.24,5.70]	[0.75,1.27]	[0.72,1.31]	[0.96,1.49]	[0.87,1.29]	[0.74,2.50]	[0.74,2.08]	[0.57,1.32]	[0.92,1.43]
40≤age<45	1.18	1.28	1.25	0.80	1.67^{**}	1.10	2.80	1.68	0.83	1.56^{**}
	[0.95,1.46]	[0.12,13.67]	[0.09,16.5]	[0.07,9.0]	[1.04,2.69]	[0.81,1.51]	[0.73,10.77]	[0.74,3.80]	[0.00,821]	[1.03,2.38]
45≤age<50	1.94^{***}	1.22	4.02	1.46	3.24^{*}	1.39	16.75	2.12	1.43	4.24^{***}
	[1.26,2.99]	[0.18,8.46]	[0.13,127.5]	[0.26,81.1]	[0.92,11.5]	[0.49,3.9]	[0.91,306.9]	[0.36,12.5]	[0.00,803346]	[1.93,9.33]
N	79 366	7 015	5 362	12 059	17 161	19 329	3 469	4 735	13 314	18 725

This table reports odds ratios from a conditional logistic regression model containing a family-specific term that captures the genetic factors common to children of the given mother–father pair. All regressions control for child's year of birth. 95 % confidence interval (CI) in brackets, adjusted for multiple hypothesis testing using the false discovery rate method. * $p < 0.1$, ** $p < 0.05$, *** $p < 0.01$. * $p < 0.1$, ** $p < 0.05$, *** $p < 0.01$ (adjusted for multiple hypothesis testing using the false discovery rate method). The excluded category is average parental age ∈ [30,34]. "Any" in the first column is at least one malformation. ICD8 categories: 746 = Congenital anomalies of heart, 749 = Cleft palate and cleft lip, 752 = Congenital anomalies of genital organs, 754 = Clubfoot (congenital), 755 = Other congenital anomalies of limbs, 756 = Other congenital anomalies of musculoskeletal system, 759 = Congenital syndromes affecting multiple systems. Infant mortality defined as stillborn or dead within 28 days of birth.

Appendix 2. Diagram of the sibling design model

Here we illustrate the sibling design model and motivate why it is preferable to a simple cohort analysis. We start with a diagram of the cohort analysis to illustrate why such a model is likely to downward bias the effect of parental age on congenital anomalies. The simple cohort analysis compares the incidence of birth defects among births with varying parental age. However, if people with different predispositions for having children with birth defects select into having children at different parental age, genetic variables and environmental factors will be important confounders, as illustrated in Panel A of Fig. 7.[1] If parents with lower predispositions for birth defects in their children (genetic variables and environmental factors less conducive of birth defects) select into older parental ages, the excluded confounders in the cohort analysis will downward bias the estimated relationship between parental age and birth defects.

We use a sibling design to deal with the concern of confounders. By comparing the differences in parental age and birth defects within siblings, we are effectively holding fixed many genetic and environmental factors which could affect the likelihood of birth defects, as illustrated in Panel B of Fig. 7. We capture these common confounders by including a family fixed effect in the regression models. The identifying assumptions are that the causal effect of parental age on birth defects is the same for all siblings within a mother-father pair, and that the causal effect of the unobserved confounders are the same for all siblings.

Fig. 7
Diagram of the cohort analysis and sibling design models

(A) Cohort analysis **(B)** Sibling design / family fixed effects

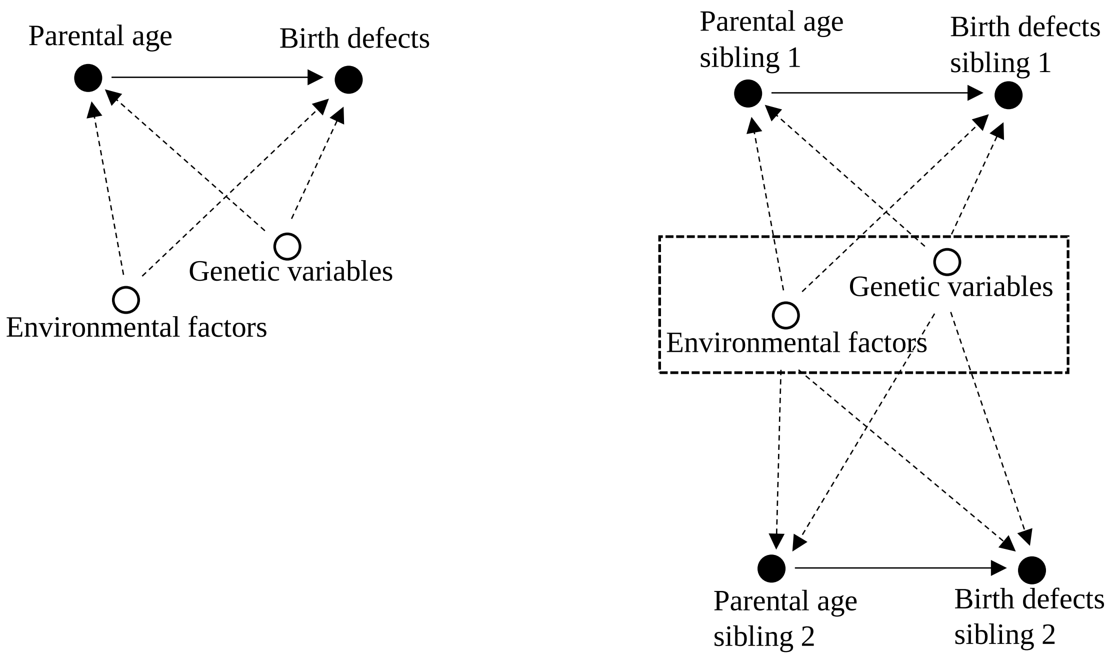

[1] A confounder is a common cause of the predictor and the outcome variable which will bias the observed association between the predictor and the outcome variable if not included in the regression.

Appendix 3. Stata syntax for main results (Table 2)

```
*** Table 2, FE-OLS results (bins) ***

/* 1. Specifying outcome variables
*/
global y "CongenitalMalformation InfantMortality"

/* 2. Running OLS-FE regressions with age categories as main predictor variables,
controlling for family and year of birth fixed effects, clustering SEs at the
family level. Store regression results
*/
foreach y in $y {
xi: quietly reghdfe `y’ age20_24 age25_29 age35_39 age40_44 age45_49, ///
vce(cluster familypid) absorb(faar familypid)
       parmest, label format(p %1.4g) saving(“`y’.dta", replace)
       }

/* 3. Adjust CIs and p-values from FE-OLS for multiple hypothesis testing
*/
foreach y in $y {
* Step 3.1. Load output data from FE-OLS regression, and keep info of interest
use "`y’.dta", clear
keep if inlist(parm, "age20_24", "age25_29" "age35_39" "age40_44" "age45_49"
keep p estimate parm

* Step 3.2. adjust p-value for multiple hypothesis testing
qqvalue p, method(hochberg) qvalue(q)
drop p

* Step 3.3. create CI adjusted for multiple hypothesis testing
gen z  = -0.862 + sqrt(0.743 - 2.404*ln(q))
gen se = abs(estimate)/abs(z)
gen min95 = estimate - (1.96*se)
max95 = estimate + (1.96*se)

* Step 3.4. Display coefficients and CI + p-value adjusted for mult. hyp. testing
order parm estimate min95 max95 q
sort parm
di “`y’”
list
}
```

```
*** Table 2, regression spline results (bins) ***

/* 1. Specifying outcome variables
*/
global y "CongenitalMalformation InfantMortality"

/* 2. Define knots at average parental age = 25, 30, 35, 40, and 45
*/
mkspline age20_24 25 age25_29 30 age30_34 35 age35_39 40 age40_44 45 age45_49 = age
```

```
/* 3. Running least square regressions with linear average parental age as the main
predictor variable, allowing for separate linear relationships within each bin
separated by the knots defined above. Controlling for family and year of birth
fixed effects, clustering SEs at the family level.
*/
foreach y in $y {
xi: quietly reghdfe `y' age20_24 age25_29 age30_34 age35_39 age40_44 age45_49, ///
vce(cluster familypid) absorb(faar familypid)
est store `y'
}

/* 4. Display results
*/
esttab CongenitalMalformation InfantMortality, ///
keep(age20_24 age25_29 age30_34 age35_39 age40_44 age45_49) ///
star(* 0.1 ** 0.05 *** 0.01) ///
cells(b(star fmt(4)) ci(par fmt(4)) p(par([ ]) fmt(4))) ///
mtitles("CongenitalMalformation " " InfantMortality ")
```
